# 3,9-Dichloro-2,4,8,10-tetra­oxa-3,9-di­phosphaspiro­[5.5]undecane-3,9-dione

**DOI:** 10.1107/S1600536810043333

**Published:** 2010-10-31

**Authors:** Zhao-Shun Zhan, Hong Wang, Li-Ping Ding, Chun-Mei Dong, Cai-Ying Sun

**Affiliations:** a Heilongjiang Key Laboratory of Molecular Design and Preparation of Flame Retardant Materials, College of Science, Northeast Forestry University, Harbin 150040, People’s Republic of China

## Abstract

In the title compound, C_5_H_8_Cl_2_O_6_P_2_, the two six-membered rings display chair conformations. The P=O bond distances are 1.444 (2) and 1.446 (2) Å. Weak inter­molecular C—H⋯O hydrogen bonds are present in the crystal structure.

## Related literature

For applications of penta­erythritol diphospho­nate compounds, see: Granzow (1981 ▶); Tanabe *et al.* (2005 ▶). For details of the preparation of the title compound, see: Li *et al.* (2002 ▶). For related compounds, see: Heinemann *et al.* (1994 ▶); Zhang *et al.* (2006 ▶). For bond-length, see: Allen *et al.* (1987 ▶); Elnagar *et al.* (2000 ▶).
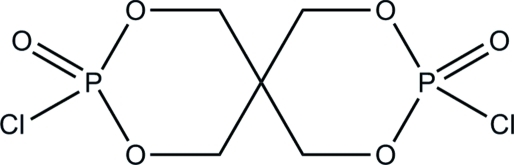


## Experimental

### 

#### Crystal data

C_5_H_8_Cl_2_O_6_P_2_
*M* *_r_* = 296.95
Orthorhombic, 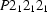

*a* = 6.0630 (5) Å
*b* = 12.7384 (10) Å
*c* = 13.4338 (10) Å
*V* = 1037.53 (14) Å^3^
*Z* = 4
Mo *K*α radiation
μ = 0.94 mm^−1^
*T* = 185 K
0.12 × 0.10 × 0.08 mm

#### Data collection

Bruker SMART CCD 1000 area-detector diffractometer
Absorption correction: multi-scan (*SADABS*; Bruker, 2001 ▶) *T*_min_ = 0.896, *T*_max_ = 0.929
5317 measured reflections
1849 independent reflections
1662 reflections with *I* > 2σ(*I*)
*R*_int_ = 0.036

#### Refinement

*R*[*F* ^2^ > 2σ(*F* ^2^)] = 0.031
*wR*(*F* ^2^) = 0.069
*S* = 1.00
1849 reflections
136 parameters
H-atom parameters constrained
Δρ_max_ = 0.27 e Å^−3^
Δρ_min_ = −0.23 e Å^−3^
Absolute structure: Flack (1983 ▶), 747 Friedel pairs
Flack parameter: 0.18 (10)

### 

Data collection: *SMART* (Bruker, 2007 ▶); cell refinement: *SAINT* (Bruker, 2007 ▶); data reduction: *SAINT*; program(s) used to solve structure: *SHELXTL* (Sheldrick, 2008 ▶); program(s) used to refine structure: *SHELXTL*; molecular graphics: *SHELXTL*; software used to prepare material for publication: *SHELXTL*.

## Supplementary Material

Crystal structure: contains datablocks I, global. DOI: 10.1107/S1600536810043333/xu5060sup1.cif

Structure factors: contains datablocks I. DOI: 10.1107/S1600536810043333/xu5060Isup2.hkl

Additional supplementary materials:  crystallographic information; 3D view; checkCIF report

## Figures and Tables

**Table 1:** Hydrogen-bond geometry (Å, °)

*D*—H⋯*A*	*D*—H	H⋯*A*	*D*⋯*A*	*D*—H⋯*A*
C1—H1*A*⋯O5^i^	0.99	2.34	3.214 (4)	147
C1—H1*B*⋯O6^ii^	0.99	2.31	3.252 (4)	159
C4—H4*B*⋯O5^i^	0.99	2.36	3.260 (4)	150

Symmetry codes: (i) 
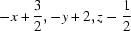
; (ii) 
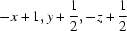
.

**Fig. 1.**
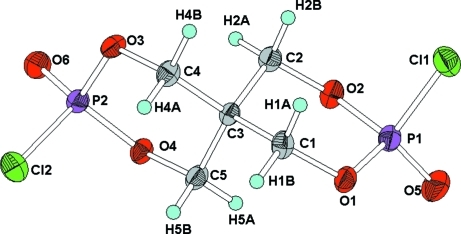
A view of the title compound, showing the atomic numbering scheme. Displacement ellipsoids are drawn at the 30% probability level.

**Fig. 2.**
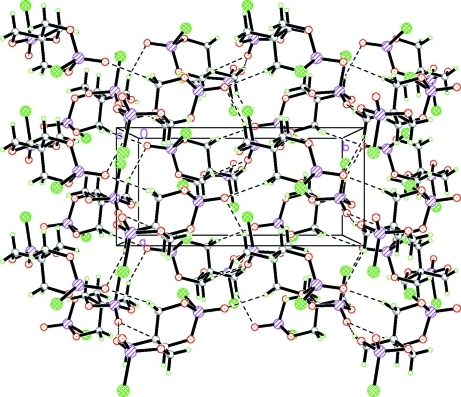
The crystal packing through C—H···O interactions along the c axis

## supplementary crystallographic information

### Comment

Studies of pentaerythritol diphosphonate compounds have been significant interested. On one hand, the compounds have been reported to act as one of the most important reaction intermediates of fire retardant agents (Tanabe *et al.*, 2005). On the other hand, it seems to be a good candidate in modifying the stability of polymers (Granzow, 1981). The findings have triggered the development of new flame retardant materials. As an extension of the work on the structural characterization of pentaerythritol diphosphonate compounds, the preparation and crystal structure of the title compound, (I), is proposed here.

The asymmetric unit of (I) contain a spiro[5.5]undecane molecule (Fig. 1). Several compounds with similar structures have been reported previously (Heinemann *et al.*, 1994; Zhang *et al.*, 2006). The bond lengths and angles are within normal ranges (Allen *et al.*, 1987; Elnagar *et al.*, 2000). The six-membered rings of (I) have the chair conformation consistent with the steric difference in this conformation between opposite ends of the molecule. In addition, the C1—C3—C2 and C4—C3—C5 angles are in the range of 109.4 (3)–109.2 (3)°,the P—Cl bond lengths are 2.0050 (14) and 2.0047 (13) Å, respectively. In the crystal structure of (I), The non-classic C—H···O hydrogen bonds ranging from 3.099 (4) to 3.260 (4) Å contributed to the stability of the crystal packing.

### Experimental

The title compound was prepared by reaction of pentaerythritol with phosphorus oxychloride in acetonitrile according to the reported procedures (Li *et al.*, 2002). Crystals were produced at the bottom of the vessel on slow evaporation of acetic acid solution.

### Refinement

All H atoms were placed geometrically with C—H = 0.99 Å and refined using a riding atom model with U_iso_(H) = 1.2U_eq_(C).

### Crystal data

**Table:**

C_5_H_8_Cl_2_O_6_P_2_	*F*(000) = 600
*M**_r_* = 296.95	*D*_x_ = 1.901 Mg m^−^^3^
Orthorhombic, *P*2_1_2_1_2_1_	Mo *K*α radiation, λ = 0.71073 Å
Hall symbol: P 2ac 2ab	Cell parameters from 2823 reflections
*a* = 6.0630 (5) Å	θ = 2.2–25.0°
*b* = 12.7384 (10) Å	µ = 0.94 mm^−^^1^
*c* = 13.4338 (10) Å	*T* = 185 K
*V* = 1037.53 (14) Å^3^	Block, colorless
*Z* = 4	0.12 × 0.10 × 0.08 mm

### Data collection

**Table:**

Bruker SMART CCD 1000 area-detector diffractometer	1849 independent reflections
Radiation source: fine-focus sealed tube	1662 reflections with *I* > 2σ(*I*)
graphite	*R*_int_ = 0.036
φ and ω scans	θ_max_ = 25.1°, θ_min_ = 2.2°
Absorption correction: multi-scan (*SADABS*; Bruker, 2001)	*h* = −5→7
*T*_min_ = 0.896, *T*_max_ = 0.929	*k* = −14→15
5317 measured reflections	*l* = −14→16

### Refinement

**Table:**

Refinement on *F*^2^	Secondary atom site location: difference Fourier map
Least-squares matrix: full	Hydrogen site location: inferred from neighbouring sites
*R*[*F*^2^ > 2σ(*F*^2^)] = 0.031	H-atom parameters constrained
*wR*(*F*^2^) = 0.069	*w* = 1/[σ^2^(*F*_o_^2^) + (0.0283*P*)^2^ + 0.5846*P*] where *P* = (*F*_o_^2^ + 2*F*_c_^2^)/3
*S* = 1.00	(Δ/σ)_max_ < 0.001
1849 reflections	Δρ_max_ = 0.27 e Å^−^^3^
136 parameters	Δρ_min_ = −0.23 e Å^−^^3^
0 restraints	Absolute structure: Flack (1983), 747 Friedel pairs
Primary atom site location: structure-invariant direct methods	Flack parameter: 0.18 (10)

### Special details

**Table:**

Geometry. All e.s.d.'s (except the e.s.d. in the dihedral angle between two l.s. planes) are estimated using the full covariance matrix. The cell e.s.d.'s are taken into account individually in the estimation of e.s.d.'s in distances, angles and torsion angles; correlations between e.s.d.'s in cell parameters are only used when they are defined by crystal symmetry. An approximate (isotropic) treatment of cell e.s.d.'s is used for estimating e.s.d.'s involving l.s. planes.
Refinement. Refinement of *F*^2^ against ALL reflections. The weighted *R*-factor *wR* and goodness of fit *S* are based on *F*^2^, conventional *R*-factors *R* are based on *F*, with *F* set to zero for negative *F*^2^. The threshold expression of *F*^2^ > σ(*F*^2^) is used only for calculating *R*-factors(gt) *etc*. and is not relevant to the choice of reflections for refinement. *R*-factors based on *F*^2^ are statistically about twice as large as those based on *F*, and *R*- factors based on ALL data will be even larger.

### Fractional atomic coordinates and isotropic or equivalent isotropic displacement parameters (Å^2^)

**Table:**

	*x*	*y*	*z*	*U*_iso_*/*U*_eq_	
P2	0.86704 (16)	0.68037 (6)	0.19061 (7)	0.0195 (2)	
P1	0.61011 (17)	0.94037 (7)	0.51917 (6)	0.0225 (2)	
Cl2	1.00827 (16)	0.75305 (7)	0.07460 (7)	0.0322 (2)	
Cl1	0.28767 (15)	0.96781 (7)	0.53971 (7)	0.0299 (2)	
O4	0.9665 (4)	0.73565 (16)	0.28413 (16)	0.0197 (5)	
O2	0.6224 (4)	0.82146 (16)	0.48872 (15)	0.0218 (5)	
O6	0.8993 (4)	0.56810 (17)	0.18897 (18)	0.0274 (6)	
O3	0.6190 (4)	0.71416 (16)	0.18620 (16)	0.0206 (5)	
O1	0.6715 (4)	1.00446 (16)	0.42462 (16)	0.0227 (6)	
O5	0.7431 (5)	0.96565 (19)	0.60521 (16)	0.0338 (6)	
C3	0.6534 (6)	0.8561 (2)	0.3088 (2)	0.0170 (7)	
C5	0.9032 (6)	0.8438 (2)	0.3065 (3)	0.0208 (8)	
H5A	0.9656	0.8912	0.2553	0.025*	
H5B	0.9652	0.8644	0.3718	0.025*	
C4	0.5581 (6)	0.8229 (2)	0.2084 (2)	0.0198 (8)	
H4A	0.3955	0.8295	0.2098	0.024*	
H4B	0.6152	0.8697	0.1555	0.024*	
C1	0.5966 (6)	0.9720 (2)	0.3254 (2)	0.0211 (7)	
H1A	0.6694	1.0155	0.2740	0.025*	
H1B	0.4353	0.9822	0.3198	0.025*	
C2	0.5455 (6)	0.7897 (3)	0.3905 (2)	0.0207 (8)	
H2A	0.3833	0.7978	0.3870	0.025*	
H2B	0.5810	0.7147	0.3796	0.025*	

### Atomic displacement parameters (Å^2^)

**Table:**

	*U*^11^	*U*^22^	*U*^33^	*U*^12^	*U*^13^	*U*^23^
P2	0.0192 (5)	0.0181 (4)	0.0211 (5)	−0.0002 (4)	0.0003 (4)	−0.0033 (4)
P1	0.0263 (5)	0.0223 (5)	0.0189 (5)	−0.0018 (4)	0.0020 (4)	−0.0028 (4)
Cl2	0.0331 (5)	0.0376 (5)	0.0259 (5)	−0.0039 (5)	0.0073 (4)	0.0002 (4)
Cl1	0.0282 (5)	0.0299 (5)	0.0317 (5)	0.0023 (4)	0.0074 (4)	−0.0002 (4)
O4	0.0187 (13)	0.0183 (12)	0.0221 (12)	0.0044 (11)	−0.0028 (10)	−0.0046 (10)
O2	0.0295 (13)	0.0182 (11)	0.0178 (12)	0.0025 (11)	−0.0017 (11)	0.0003 (9)
O6	0.0283 (14)	0.0192 (12)	0.0346 (14)	0.0004 (11)	0.0046 (13)	−0.0042 (11)
O3	0.0205 (12)	0.0169 (11)	0.0245 (12)	−0.0013 (10)	−0.0035 (12)	−0.0037 (10)
O1	0.0307 (15)	0.0169 (11)	0.0206 (12)	−0.0025 (11)	0.0042 (11)	−0.0037 (10)
O5	0.0393 (16)	0.0398 (15)	0.0223 (12)	−0.0063 (14)	−0.0051 (13)	−0.0065 (11)
C3	0.0198 (18)	0.0146 (15)	0.0166 (16)	0.0006 (13)	−0.0007 (15)	−0.0015 (13)
C5	0.0248 (19)	0.0164 (16)	0.0212 (18)	−0.0009 (15)	−0.0002 (17)	−0.0046 (14)
C4	0.0219 (19)	0.0151 (16)	0.0223 (19)	0.0042 (15)	−0.0024 (15)	−0.0015 (14)
C1	0.028 (2)	0.0183 (17)	0.0167 (17)	0.0027 (16)	0.0007 (16)	0.0000 (14)
C2	0.024 (2)	0.0189 (18)	0.0196 (18)	0.0002 (15)	0.0002 (15)	−0.0014 (14)

### Geometric parameters (Å, °)

**Table:**

P2—O6	1.444 (2)	C3—C5	1.523 (5)
P2—O4	1.561 (2)	C3—C4	1.528 (4)
P2—O3	1.565 (2)	C3—C2	1.532 (4)
P2—Cl2	2.0047 (13)	C3—C1	1.533 (4)
P1—O5	1.446 (2)	C5—H5A	0.9900
P1—O1	1.555 (2)	C5—H5B	0.9900
P1—O2	1.571 (2)	C4—H4A	0.9900
P1—Cl1	2.0050 (14)	C4—H4B	0.9900
O4—C5	1.461 (3)	C1—H1A	0.9900
O2—C2	1.457 (4)	C1—H1B	0.9900
O3—C4	1.464 (4)	C2—H2A	0.9900
O1—C1	1.467 (4)	C2—H2B	0.9900
			
O6—P2—O4	114.00 (13)	O4—C5—H5A	109.4
O6—P2—O3	113.70 (14)	C3—C5—H5A	109.4
O4—P2—O3	106.12 (13)	O4—C5—H5B	109.4
O6—P2—Cl2	112.82 (11)	C3—C5—H5B	109.4
O4—P2—Cl2	104.61 (10)	H5A—C5—H5B	108.0
O3—P2—Cl2	104.71 (10)	O3—C4—C3	110.2 (3)
O5—P1—O1	113.75 (14)	O3—C4—H4A	109.6
O5—P1—O2	113.36 (14)	C3—C4—H4A	109.6
O1—P1—O2	106.39 (12)	O3—C4—H4B	109.6
O5—P1—Cl1	113.26 (12)	C3—C4—H4B	109.6
O1—P1—Cl1	104.73 (10)	H4A—C4—H4B	108.1
O2—P1—Cl1	104.49 (11)	O1—C1—C3	109.5 (2)
C5—O4—P2	119.3 (2)	O1—C1—H1A	109.8
C2—O2—P1	119.24 (19)	C3—C1—H1A	109.8
C4—O3—P2	119.6 (2)	O1—C1—H1B	109.8
C1—O1—P1	121.3 (2)	C3—C1—H1B	109.8
C5—C3—C4	109.2 (3)	H1A—C1—H1B	108.2
C5—C3—C2	112.5 (3)	O2—C2—C3	111.0 (3)
C4—C3—C2	108.6 (3)	O2—C2—H2A	109.4
C5—C3—C1	109.0 (3)	C3—C2—H2A	109.4
C4—C3—C1	108.0 (3)	O2—C2—H2B	109.4
C2—C3—C1	109.4 (3)	C3—C2—H2B	109.4
O4—C5—C3	111.2 (3)	H2A—C2—H2B	108.0

### Hydrogen-bond geometry (Å, °)

**Table:**

*D*—H···*A*	*D*—H	H···*A*	*D*···*A*	*D*—H···*A*
C1—H1A···O5^i^	0.99	2.34	3.214 (4)	147
C1—H1B···O6^ii^	0.99	2.31	3.252 (4)	159
C4—H4B···O5^i^	0.99	2.36	3.260 (4)	150

Symmetry codes: (i) −*x*+3/2, −*y*+2, *z*−1/2; (ii) −*x*+1, *y*+1/2, −*z*+1/2.
